# Determination of imipenem efflux-mediated resistance in *Acinetobacter spp.*, using an efflux pump inhibitor

**Published:** 2019-10

**Authors:** Ghazale Amiri, Maryam Abbasi Shaye, Masoumeh Bahreini, Asghar Mafinezhad, Kiarash Ghazvini, Mohammad Reza Sharifmoghadam

**Affiliations:** 1 Deparment of Biology, Faculty of Science, Ferdowsi University of Mashhad, Mashhad, Iran; 2 Department of Microbiology, Shahid Kamyab Hospital, Mashhad University of Medical Sciences, Mashhad, Iran; 3 Department of Microbiology, School of Medicine, Mashhad University of Medical Sciences, Mashhad, Iran

**Keywords:** *Acinetobacter baumannii*, *Acinetobacter lwoffii*, Imipenem, Antibiotic resistance, Efflux pumps

## Abstract

**Background and Objectives:**

In recent years, reports of *Acinetobacter* strains resistant to all known antibiotics have caused a great concern in medical communities. Overexpression of efflux pumps is one of the major causes of resistance in bacteria. The aim of this study was to investigate the role of efflux pumps in conferring resistance to imipenem in clinically important *Acinetobacter spp; Acinetobacter baumannii* and *Acinetobacter lwoffii*.

**Materials and Methods:**

A total number of 46 clinical *Acinetobacter* isolates, including 33 *A. baumannii* and 13 *A. lwoffii* isolates, previously collected from Shahid Kamyab and Ghaem hospitals of Mashhad, Iran were used in this study. Imipenem susceptibility testing was carried out by the disc diffusion method. Imipenem minimum inhibitory concentration (MIC) for resistant *Acinetobacter* isolates were determined both in the presence and absence of the efflux pumps inhibitor, carbonyl cyanide 3-chlorophenylhydrazone (CCCP).

**Results:**

Resistance to imipenem was observed in 38 isolates including 30 *A. baumannii* and 8 *A. lwoffii* isolates. Experiments in the presence of CCCP showed a 2 to 16384 fold reduction in imipenem MICs in 14 *A. baumannii* and 2 *A. lwoffii* isolates.

**Conclusion:**

The results obtained showed high levels of resistance to imipenem and contribution of efflux pumps in conferring resistance in both *Acinetobacter* species in this study. Moreover, imipenem efflux mediated resistance highlights the importance of this mechanism not only in *A. baumannii* but also in non-*baumannii Acinetobacter* Spp. which have been neglected in antibiotic resistance studies.

## INTRODUCTION

Drug resistance is the biological and natural phenomenon of bacteria in response to the selective pressure of an antimicrobial agent (1). Nowadays, drug resistance is threatening the global public health and causing many costly clinical problems, especially in developing countries (2). Drug resistance first appears in hospitals, where most antibiotics are used. Every year, millions of kilograms of antibiotics are used worldwide to prevent and treat infections in people and animals. Increasing use of antimicrobial drugs, especially in developing countries, where antibiotics are readily available and often over-used or misused, have led to higher levels of resistance in different bacteria (2, 3). *Acinetobacter baumannii*, the most important species of *Acinetobacter* genus, is one of the major pathogens for medical institutions around the world. Over the past 15 years, the clinical significance of *A. baumannii* has increased because of its considerable ability to obtain resistance factors, which has turned it into one of the most threatening organisms in today’s world (4). The extensive resistance of *Acinetobacter* to antimicrobial agents is probably due to its relatively impermeable outer membrane and its exposure to a large reservoir of resistance genes in the environment (5). *Acinetobacter* can receive resistance genes from other organisms. The mutations that result in resistance can spread over time or become dominant in the prevailing populations under the selective antibiotic pressure conditions (6).

Carbapenems (imipenem, meropenem, ertapenem and doripenem) are broad-spectrum antibiotics as well as the most effective antibiotics of β-lactams that are effective against most microorganisms and often used as the last resort for the treatment of cephalosporin-resistant infections (7). In recent years, reports of resistance in *A. baumannii* isolates to all known antibiotics including carbapenems have increased, which have been of great concern in medical communities (4). One of the mechanisms involved in resistance in *Acinetobacter spp.* is the over expression of efflux pumps. Bacteria uses efflux pumps to excrete extracellular toxic substances (drugs, chemicals and antibiotics) that penetrate into the cell, which both helps the bacteria to survive in conditions of environmental stress and to prevent from formation of an effective antibiotic concentration to act (8). Moreover, efflux pumps give the bacteria the chance to equip itself to other resistance mechanisms while helping it to survive in antibiotic stress (9). Efflux pumps are present in all living organisms (prokaryotes and eukaryotes) and keep them away from the toxic effects of organic chemicals. Multidrug resistance in bacteria is often accompanied by overexpression of these transmitters. Efflux pumps usually act synergistically with decreasing the permeability of the outer membrane, which leads to removing antibacterial agents (8). Efflux pumps are divided into 5 families based on the amino acid sequences, energy source, subunit number, folding and their substrates, which are ABCs (ATP-binding Cassette) that use ATP energy to transfer substrates and RND (Resistance-nodulation-cell division), SMR (Small multidrug resistance), MFS (Major Facilitator Superfamily) and MATE (Multidrug and Toxic compound Extrusion) that use Proton gradient as the source of energy for transferring substrates (8). The overexpression of efflux pumps can be assessed using Efflux Pump Inhibitors (EPIs) such as reserpine, valinomycin, dinitrophenol (DNP), carbonyl cyanide 3-chlorophenylhydrazone (CCCP) and phenylalanine-arginine β-naphthylamide (PaβN). An increase in antibiotic susceptibility in the presence of these inhibitors can indicate the role of efflux pumps in causing resistance in bacteria. However, EPIs are only used to assess the activity of efflux pumps in the lab and they cannot be used in clinical and therapeutic applications because of their toxicity and interference with other cellular functions (10).

The aim of the present study is to investigate the role of efflux pumps in imipenem resistance in clinical isolates of *Acinetobacter spp.*

## MATERIALS AND METHODS

### Bacterial strains

Forty-six *Acinetobacter spp.* clinical isolates, including 33 *Acinetobacter baumannii* and 13 *Acinetobacter lwoffii* were investigated in this study. They were isolated from patients hospitalized at Shahid Kamyab and Ghaem Hospitals in Mashhad, Iran and identified using GNA Kit (Microgen, United Kingdom) (11). The ethics committee of Ferdowsi University of Mashhad approved the design and protocol of the study.

### Antibiotic susceptibility tests

Imipenem susceptibility testing was done using disk diffusion test according to Clinical and Laboratory Standards Institute (CLSI) protocols (12). The imipenem disc (10 μg) was purchased from HiMedia Company, India.

### Determination of Minimum inhibitory concentration (MIC)

The MICs of imipenem was determined in a range of 1 to 512 μg/ml for all imipenem resistant *Acinetobacter* isolates. Determination of imipenem MIC was repeated in the presence of efflux pumps inhibitor, cyanide 3-chlorophenylhydrazone (CCCP), in order to investigate the role of efflux pumps in imipenem resistant isolates. To determine the appropriate concentration of CCCP, an MIC determination of CCCP was also carried out and a concentration equals to 1/2 and 1/4 of the determined MIC was used as non-inhibitory concentration. Determination of MIC values was carried out using the broth microdilution method in 96 well micro plates according to CLSI standards (12). Imipenem and cyanide 3-chlorophenylhydrazone powder were purchased from Sigma Aldrich, United States of America.

## RESULTS

### Antibiotic susceptibility tests

Resistance to imipenem was observed in 38 isolates (82.6%) including 30 *A. baumannii* (out of the total number of 33 *A. baumannii* isolates) and 8 *A. lwoffii* (out of the total number of 13 *A. lwoffii* isolates).

### Determination of minimum inhibitory concentration (MIC)

Determination of CCCP MIC was conducted for each of the imipenem-resistant *Acinetobacter* isolates. It ranged from 0.39 to 50 μg/ml among 38 *Acinetobacter* isolates which in most cases (25 isolates); it was 25 – 50 μg/ml. Imipenem MIC was also determined for each 38 imipenem resistant isolates alone and in the presence of appropriate concentration of CCCP. High levels of resistance to imipenem (32 to 512 μg/ml) was observed in all resistant *Acinetobacter* isolates.

Overall, 16 isolates including 14 out of 30 resistant isolates of *A. baumannii*, and 2 out of 8 resistant isolates of *A. lwoffii* showed a 2 to 16384 folds reduction in imipenem MIC in the presence of CCCP. Table 1 shows imipenem MIC values in the presence and absence of two different CCCP concentrations in the 16 resistant *Acinetobacter* isolates.

**Table 1:** Imipenem MIC values of resistant isolates in the presence and absence of two different concentrations of CCCP.

**Number**	**Species**	**MIC of Imipenem (μg/ml)**	**MIC of Imipenem in presence of CCCP (1/2) MIC (μg/ml)**	**Reduction in MIC of Imipenem**	**MIC of Imipenem in presence of CCCP (1/4) MIC (μg/ml)**	**Reduction in MIC of Imipenem**
5^*^	*A. baumannii*	512	256	1/2	256	1/2
10^*^	*A. baumannii*	256	128	1/2	256	No Change
12^*^	*A. baumannii*	512	256	1/2	256	1/2
13^*^	*A. baumannii*	128	64	1/2	128	No Change
14^***^	*A. baumannii*	256	0.25	1/1024	256	No Change
16^*^	*A. baumannii*	32	16	1/2	16	1/2
19^***^	*A. baumannii*	256	0.016	1/16384	64	1/4
25^**^	*A. baumannii*	256	2	1/128	2	1/128
27^**^	*A. baumannii*	128	2	1/64	128	No Change
28^**^	*A. baumannii*	128	4	1/32	4	1/32
31^**^	*A. baumannii*	128	8	1/16	8	1/16
32^**^	*A. baumannii*	128	8	1/16	8	1/16
37^*^	*A. baumannii*	512	256	1/2	256	1/2
38^***^	*A. lwoffii*	32	0.008	1/4096	32	No Change
39^***^	*A. lwoffii*	32	0.031	1/1024	2	1/16
42^*^	*A. baumannii*	512	256	1/2	256	1/2

CCCP (1/2): a concentration of CCCP equals to one half of its MIC determined for the specific isolate

CCCP (1/4): a concentration of CCCP equals to a quarter of its MIC determined for the specific isolate

* Isolates with mild reduction in imipenem MIC in the presence of CCCP

** Isolates with high levels of reduction in imipenem MIC in the presence of CCCP

*** Isolates with extreme levels of reduction in imipenem MIC in the presence of CCCP

## DISCUSSION

In the present study, 38 isolates (30 *A. baumannii* and 8 *A. lwoffii*) showed resistance to imipenem. The MICs of imipenem among resistant isolates was 32 to 512 μg/ml. Totally, 16 isolates out of 38 resistant *Acinetobacter* isolates (42.1%) including 14 *A. baumannii* and 2 *A. lwoffii* isolates showed reduction in their imipenem MIC in the presence of efflux pump inhibitor (CCCP), indicating the involvement of efflux pumps in resistance to imipenem. The results of the antibiotic susceptibility testing and MIC determination of *Acinetobacter* isolates confirmed not only the importance of *A. baumannii* in nosocomial infections and antibiotic resistance, which is the center of attention in many researches all around the world (13, 14), but also highlight the significance of *A. lwoffii* in the same topic which has been less studied in regards with resistance patterns and mechanisms.

Resistance to imipenem was observed among 82.6% of the isolates in this study which includes 79% of all *A. baumannii* isolates (30 out of 38) and 61.5% of all *A. lwoffii* isolates (8 out of 13). According to the study done by Van Loveren et al. (2004), carbapenems including imipenem were the most effective antibiotics against *Acinetobacter* infections in European countries where 62% to 100% of the *A. baumannii* isolates and 73% to 100% of the *A. lwoffii* isolates were susceptible to imipenem (15). Imipenem resistance was reported among 97% and 78% of the studied *A. baumannii* isolates by Gholami et al. (2014) in Tehran (16), which show the increasing prevalence of resistance among *Acinetobacter* isolates over time. Moreover, the MICs of imipenem were between 32 to 512 μg/ml in the present study, which is higher comparing with the studies done by Taheri Kalani et al. (2008) who reported MICs of imipenem from 16 to 256 μg/ml in *Acinetobacter* isolates (17), which suggests that levels of imipenem resistance has also increased during the time. Among 38 imipenem resistant isolates, 16 (42.1%) showed a 2 to 16384 fold reduction in MIC in the presence of CCCP. Gholami et al. (2014) reported a 2 to 64 fold of reduction in MICs in the presence of PaβN in a similar study (16). In the study done by Hou et al. (2012) in China, 66% of the *A. baumannii* isolates showed 4 to 32 folds of reduction (18). While the proportion of the isolates showing efflux mediated resistance has been more in the study done by Hou et al. (18).

According to the results, the rate of reduction has been increasingly higher in the present study compared to previous studies. Among 16 isolates (including 14 *A. baumannii* and 2 *A. lwoffii*) showing reduction in MICs in the presence of CCCP, 7 isolates showed a mild reduction (2 to 8 folds), 8 isolates showed high levels of reduction (16 to 128 folds) and 4 isolates showed extreme levels of reduction (More than 256 folds) in MICs. Among isolates with extreme levels of reduction, 2 isolates were *A. baumannii* (Ac14 and Ac19 showed 1024 and 16384 folds of reduction in MIC, respectively) and 2 isolates were *A. lwoffii* (Ac38 and Ac39 showed 4096 and 1024 folds of reduction in MIC, respectively), which shows very high contribution of efflux pumps in conferring resistance to imipenem in both species. In the previous study on the same isolates by Abbasi Shaye et al. (2018) efflux pumps were also suggested to be mainly involved in amikacin resistance (2 to 524288 folds) (11). Generally, nine *A. baumannii* isolates of this study, which showed reduction in MICs in the presence of CCCP, were also reported as amikacin efflux mediated resistant isolates in the previous study by Abbasi Shaye et al. (11). This could be due to over expression of several types of efflux pumps at the same time or could be resulted from the fact that efflux pumps can actively export substrates of different groups (19). However, no efflux mediated resistance to amikacin was observed in *A. lwoffii* isolates in Abbasi Shaye’s study (11).

In conclusion, very high contribution of efflux pumps to imipenem resistance in *Acinetobacter* isolates indicates great importance of this mechanism in antibiotic resistance, which could rapidly spread among *A. baumannii* strains as well as other *Acinetobacter* species, more particularly *A. lwoffii*. This is the first report of imipenem efflux mediated resistance in *A. lwoffii* isolates, which is of great importance according to high ability of *Acinetobacter* species in acquiring resistance genes.
